# Development of a Standardised System to Classify Injury-Inciting Circumstances in Football: the Football Injury Inciting Circumstances Classification System (FIICCS)

**DOI:** 10.1007/s40279-023-01857-6

**Published:** 2023-05-26

**Authors:** Francesco Aiello, Alan McCall, Susan J. Brown, Andreas Serner, Lauren V. Fortington, Suzanne Afra Elisabeth Huurman, Colin Lewin, Masashi Nagao, James O’Brien, Anastasia Panossian, Ricard Pruna, Guilherme Passos Ramos, Matthew Whalan, Franco M. Impellizzeri

**Affiliations:** 1 Arsenal Performance and Research Team, Arsenal Football Club, London, UK; 2 School of Applied Sciences, Edinburgh Napier University, Edinburgh, UK; 3 FIFA Medical, Fédération Internationale de Football Association, Zurich, Switzerland; 4 School of Medical and Health Sciences, Edith Cowan University, Joondalup, Australia; 5 Medical Department Real Madrid CF, Madrid, Spain; 6 Sports Medicine Department, St Antonius Hospital, Utrecht, The Netherlands; 7 The Lewin Sports Injury Clinic, East London, UK; 8 Medical Technology Innovation Center, Juntendo University, Bunkyo-ku, Tokyo, Japan; 9 Department of Orthopaedic Surgery, Juntendo University Faculty of Medicine, Bunkyo-ku, Tokyo, Japan; 10 Department of Sports Medicine, Juntendo University, Bunkyo-ku, Tokyo, Japan; 11 Red Bull Athlete Performance Center, Salzburg, Austria; 12 Chelsea FC, London, UK; 13 FC Barcelona Medical Services, Barcelona, Spain; 14 Brazilian Football Confederation (CBF), Rio de Janeiro, RJ Brazil; 15 Endocrinology and Metabolism Laboratory, Department of Physiology and Biophysics, Federal University of Minas Gerais (UFMG), Belo Horizonte, MG Brazil; 16 Centre of Medical and Exercise Physiology, School of Medicine, University of Wollongong, Wollongong, NSW Australia; 17 Football Australia, Sydney, Australia; 18 Faculty of Health, Sport and Exercise Discipline Group, University of Technology Sydney, Sydney, Australia

## Abstract

**Background:**

A comprehensive examination of the sport-specific activities and circumstances being performed at the time of injury is important to hypothesise mechanisms, develop prevention strategies and inform future investigations. Results reported in the literature are inconsistent because inciting activities are reported using different classifications. Hence the aim was to develop a standardised system for the reporting of inciting circumstances.

**Methods:**

The system was developed using a modified Nominal Group Technique. The initial panel included 12 sports practitioners and researchers from four continents with respectively ≥ 5 years of experience working in professional football and/or conducting injury research. The process consisted of six phases: idea generation, two surveys, one online meeting and two confirmations. For answers to the closed questions, consensus was deemed achieved if ≥ 70% of respondents agreed. Open-ended answers were qualitatively analysed and then introduced in subsequent phases.

**Results:**

Ten panellists completed the study. The risk of attrition bias was low. The developed system includes a comprehensive range of inciting circumstances across five domains: contact type, ball situation, physical activity, session details, contextual information. The system also distinguishes between a core set (essential reporting) and an optional set. The panel deemed all the domains to be important and easy to use both in football and in research environments.

**Conclusion:**

A system to classify inciting circumstances in football was developed. Given the extent of reporting inconsistency of inciting circumstances in the available literature, this can be used while further studies evaluate its reliability.

**Supplementary Information:**

The online version contains supplementary material available at 10.1007/s40279-023-01857-6.

## Key Points

**Table:** 

A standardised system to report the circumstances of injury in football has been developed by football and research experts.
The system can be integrated into data collection routines already implemented in football.

## Introduction

Understanding the circumstances and the activities performed at the time of injury is important to identify potential mechanisms, hypothesise causal relationships and eventually develop injury prevention strategies that can be tested [1]. Unfortunately, studies investigating injury-inciting circumstances (i.e. the circumstances during which injuries occur) have typically used different classification systems [2], making comparisons between studies difficult. This issue of inconsistent reporting is not consigned only to the sports medicine literature but is also common in the healthcare field, where it has been shown that the use of different classification systems might lead to the implementation of inadequate interventions in reducing a targeted outcome [3]. Similar inconsistency in sports medicine and football is likely and poses a similar risk of the development and implementation of prevention strategies that are not targeting appropriately.

Accurate and consistent recording and reporting of how injury occurs (i.e. the inciting circumstances or, as commonly referred to, injury mechanisms) is key to being able to combine, compare and generalise findings across studies and then provide robust information to practitioners [4].

Such consistency could be achieved using a standardised core outcome set (COS), that is, a set of outcomes and information that should always be reported as a minimum requirement. The use of a standardised COS would allow studies to be performed that are homogeneous but would still allow researchers and practitioners to collect and report additional outcomes or information if they wish to do so (i.e. offering the possibility of recording optional standard and self-defined outcome sets beyond the core set) [3, 5]. Furthermore, using a standardised classification system would reduce the risk of outcome-reporting bias (i.e. the reporting of only a subset of the outcomes) [6]. These are the goals of initiatives such as the Core Outcome Measures in Effectiveness Trials (COMET) [3].

Guidelines for injury reporting (e.g. type, severity) have been published, and it has been recommended to develop sport-specific guidelines for the reporting of inciting circumstances [7]. A classification system to describe the injury-inciting circumstances in football named “football incident analysis” was developed by Andersen et al. [8], but this system has been rarely used since its development in 2003. We postulate that this may be in part due to the time needed to report all the information included in this classification system or perhaps because the relevant stakeholders who should use it (e.g. football practitioners working with teams) were not involved in the development of the system [9, 10]. Given the continued importance of injury prevention and lack of standardised injury classification system, having a reference classification system consisting of a COS with additional options would allow practitioners and researchers to consistently collect and report data concerning inciting circumstances, which can guide research around injury mechanisms and the development of injury prevention strategies.

Therefore, the aims of this study were: (1) to develop a core outcome set that can be implemented in practice and research to classify the injury-inciting circumstances, and (2) to develop an optional (additional) outcome set not too time demanding but that allows the reporting of other relevant information around the inciting circumstances.

## Methods

The study was conducted following the COMET handbook guidelines [3]. Ethical approval was granted by Edinburgh Napier University’s School of Applied Sciences Research Integrity Committee (SAS/2773451) and panellists provided electronic consent prior to participation.

### Study Protocol

To ensure that a wide range of knowledge and experience was considered in the decision-making process and to increase the possibility that the decisions will have an impact on future policies and practices, the Football Injury Inciting Circumstances Classification System (FIICCS) was developed involving a wide range of stakeholders [3, 9]. For the aim of this study, the stakeholders deemed as most appropriate were (in no particular order of relevance) sports medicine doctors, physiotherapists, sport scientists, strength and conditioning (S&C) coaches, and sport science/medicine researchers, with experience in the collection of injury data in the practical setting and/or use of such data for research purposes. A modified nominal group technique (NGT) model was used. NGT models can be modified and adapted depending on the context in which they are implemented as well as to account for participants’ time and to allow panellists to meet, interact and discuss divergent views. This could lead the group to consider different options, which is one of the main advantages of consensus methods [9, 11, 12]. The model implemented in this study consists of six phases summarised as follows:

1. Idea generation: the steering committee, formed by four authors (FA, AM, SJB, FMI), reviewed the literature (more detailed methods outlined below) and subsequently generated a draft of the FIICCS. This follows the modified NGT model proposed by McMillan et al. [11] which was implemented as panellists were either experienced football practitioners and/or researchers and had limited time.
2. First ranking: the draft of the FIICCS developed by the steering committee was delivered to the panellists, who were required to express their opinion and to suggest improvements on the system using closed and open-ended questions.
3. Pre-meeting survey: the results of phase 2 were circulated to panellists, who were then asked to rate their agreement with the improvements suggested in the open-ended questions.
4. Panel discussion: panel members’ answers provided in phase 3 were analysed and circulated to all panellists. Subsequently, an online meeting was held to allow panellists to discuss the disagreements arising from phases 2 and 3.
5. Confirmation: after the end of the discussion, the FIICCS was updated and then circulated to the panellists, who were asked to confirm their level of agreement with the final version.
6. Alignment with FIFA Football Language and Football injury surveillance methodology consensus: the terminology of the FIICCS was aligned with FIFA Football Language [13] and with the football extension of the International Olympic Committee consensus on methods for recording and reporting of epidemiological data on injury and illness in sport (STROBE-SIIS) [14], and the panellists were asked to further confirm their agreement with this alignment.

### Phase 1: Idea Generation

During phase 1, the steering committee developed the first draft of the FIICCS. This was done through a systematic review of the literature. A systematic search was carried out, and the list of the activities which lead to injury reported in the literature was obtained from the studies included and was organised into domains and sub-domains to build the draft of the FIICCS (details of the systematic search and the list of the circumstances reported in the literature can be found in the original study [2]). Subsequently, the draft FIICCS was discussed within the steering committee for 3 months (between 10 February and 20 May 2021) until agreement was achieved. The first draft of the FIICCS included six domains: contact type, physical activity, ball situation, playing position, session detail and contextual information. Each domain was structured to include all activities reported in the literature. To increase the usability of the system the domains of the system were split into two sections: core set and optional set (Fig. S3). The core set constitutes the agreed minimum information required for reporting on the inciting circumstances in football and must always be reported. The optional set includes details that would help towards a deeper understanding of the inciting circumstances but are not deemed essential requirements. To ensure the clarity, the FIICCS was piloted by two sport scientists with experience working in professional football, who were asked to look at the structure of the system (provided in form of flow chart) and to test it using seven video clips randomly selected and provided by the steering committee. The clips showed injuries which occurred during football practice and allowed them to consider all the parts of the FIICCS. After the injuries were classified, they were asked to provide feedback on the clarity of the system, the clarity of the guidelines, how the system was being tested and recommendations to improve it. The sport scientists who piloted the system were highly qualified and had experience in collecting data and conducting research on football injuries (highest level of education: PhD 2; nationality: Italian 1, Scottish 1; years of experience working in professional football: 5 and 22). No changes were required to the FIICCS after pilot testing, while the guidelines for testing the system were updated to make the panellists aware that they could need to revisit the clips for further review of the injuries more than once. The practitioners included in the pilot group were not included in the subsequent expert panel.

### Phase 2: First Ranking

The FIICCS was circulated to the panel group. The members of the panel were recruited through purposive sampling [15] from the authors’ network and knowledge of the practitioners working in injury research and professional football. To be included, the experts had to be fluent English speakers and have at least 5 years of experience in conducting research on football injuries or 5 years of experience working in professional football with duties concerning diagnosis or prevention of injuries or return-to-play protocols after injury (Table 1). No geographical limitations were put in place, and indeed, experts were included from different global regions and backgrounds. In total, 21 football practitioners and researchers were invited to take part in the study (men *n* = 13, women *n* = 8; doctors *n* = 7, physiotherapists *n* = 4, injury researchers *n* = 2, S&C coaches *n* = 3, sport scientists *n* = 5), 15 of whom agreed to participate (men *n* = 11, women *n* = 4; doctors *n* = 6, physiotherapists *n* = 4, injury researchers *n* = 2, S&C coaches *n* = 2, sport scientists *n* = 1). Two weeks were given to potential panellists to agree to taking part in the study.

**Table 1:** Inclusion criteria for members of the panel group

Position	Experience	Duties (at least one)
Doctors, physiotherapists, S&C coaches and sport scientists	Minimum 5 years of experience working in professional football	Responsible for injury surveillance programmes AND/OR Experience with manually inputting injury data into surveillance systems AND/OR Experience on development and/or implementation of injury prevention programmes AND/OR Experience on management of return to play programmes AND/OR Experience of diagnosing injuries
Researchers	Minimum 5 years of experience conducting research on injuries in football	Research on injury prevention strategies AND/OR Research on mechanisms of injury AND/OR Research on injury reporting standards/guidelines AND/OR Research on factors associated to injury occurrence

The panellists were provided with the FIICCS in flow chart format (Fig. S3) and within an online survey. Panellists were requested to look at the structure of the FIICCS and to test it using seven video clips provided by the research team. The clips showed injuries which all occurred during football practice and allowed the panellists to consider all parts of the FIICCS. After the panellists tested the system, they were asked to answer an online survey to rate their agreement with the content and the organisation of each domain, the importance of reporting on each domain, the clarity of each domain and the difficulty of reporting on each domain in football and research environments.

The survey included five closed and seven open-ended questions and was designed to examine the content validity of the system and to ask panellists to provide recommendations on how to improve the system. The 5-point Likert scales included in the closed questions were developed using the anchors proposed by Olaoluwa [16]. The aim was to keep the duration of this phase (i.e. testing the system and answering the survey) below 40 min due to the limited availability of the panellists. To do so, the closed questions were administered for the core set and the optional set of each domain (i.e. core set of physical activity, optional set of physical activity and so on). Panellists could specify which part they did not agree with, suggest improvements using the open-ended questions (Table S1), and were free to not answer questions if they did not want to. The survey was administered using the online platform Novi Survey 8.7 (https://novisurvey.net/). The panellists were given 2 months (from 19 July 2021 to 19 September 2021) to respond to the survey. Three reminder emails were sent to panellists over the 2-month period, after which they were considered to have withdrawn if no response was received.

### Phase 3: Pre-meeting Survey

After the completion of phase 2, panellists were provided with the results of the survey and were asked to rate their agreement with the improvements suggested in the previous phase. The aim of this phase was to identify the improvements on which panellists agreed and therefore did not need to be discussed further during the meeting. This reduced the number of topics to discuss and helped shorten the time required for discussion. The survey included 30 closed questions (Table S2) and was administered using the software and Likert scales described in phase 2. Panellists were given 4 weeks (from 7 October 2021 to 4 November 2021) to respond to the survey. Two reminder e-mails were sent to panellists over the 4-week period, after which they were considered withdrawn if no response was received.

### Phase 4: Panel Discussion

After the completion of the pre-meeting survey, panellists were invited to an online meeting to discuss the topics around which consensus had not been reached. The meeting took place on 18 November 2021 on Microsoft Teams. One week before the meeting, panellists were provided with the results of the pre-meeting survey and with the list of the topics that were going to be discussed. Twelve points on which consensus was not previously reached were discussed during the meeting (Table S8).

The meeting was facilitated by F.A. and F.M.I., the latter with previous experience. At the start of the meeting, it was explained to the panellists that they were free to express their opinion and that they were not obliged to change their opinion just to reach consensus. Panellists were asked to listen and consider others’ opinions openly and respectfully. To ensure all panellists felt comfortable and free to express their opinion, the meeting was not recorded. F.A. took hand notes during the meeting.

Before beginning the discussion, panellists were asked to briefly introduce themselves. Subsequently, the first topic was introduced, and panellists were requested to discuss. No time limits were given for the discussion of each topic, albeit to account for the limited time of the panel it was agreed that the meeting would last no more than 2 h. At the end of each discussion, the panellists were asked to express their agreement with the group decision through an anonymous survey performed on Microsoft Teams. The scales for clarity and agreement were the same as those implemented in previous phases (Table S1). However, to encourage panellists to state a preference and facilitate a consensus, the neutral options “neither agree nor disagree” and “good” were removed. However, participants were allowed not to respond.

Two panellists could not make the online meeting due to last-minute commitments with their respective football teams. These two panellists were not excluded from the study but were provided with an anonymous summary of the panel discussion (based on the facilitator notes) and were invited to respond to a survey to express their agreement with the group decisions.

### Phase 5: Confirmation

Following the panel discussion, the FIICCS was updated, and the panel was asked to confirm their agreement with the final version. The final version was sent to the panel together with a report of the discussion including the results of the anonymous polls, and the panel was asked to confirm their agreement with the system and to inform of any objections. This phase took place between 13 December 2021 and 6 January 2022.

### Phase 6: Alignment with FIFA Football Language and Football Injury Surveillance Methodology Consensus

Following the initial confirmation from panellists, one panellist suggested aligning the system with the football extension of the International Olympic Committee consensus on methods for recording and reporting of epidemiological data on injury and illness in sport and with the FIFA Football Language. The FIFA Football Language defines each player’s actions within a football match and can be used to analyse players’ and teams’ actions [13]. Therefore, the activities and their descriptions were subsequently aligned and the updated version sent to the panellists who were asked if they had any objections to the amendments. This phase took place between 15 January and 10 June 2022 and was facilitated by a FIFA member.

### Data Analysis

Raw data were exported and analysed in RStudio version 1.3.1056 through the packages *beeswarm, cowplot* and *ggplot2* [17, 18, 19, 20]. The number of answers provided for each anchor (e.g. strongly agree, strongly disagree) was divided by the total number of answers provided to calculate the percentage of consensus. Such percentages are reported in figures, while the raw number of answers are reported in tables. Consensus was deemed as being achieved when at least 70% of responders reported agreement or disagreement. This cut-off was arbitrarily selected a priori by the research team following methods implemented in other similar studies [21, 22, 23] as guidelines for selection of consensus thresholds do not exist [3, 15, 24]. Nevertheless, the exact agreement values were also reported. Items on which agreement was reached were excluded from subsequent rounds and either included or excluded in the system depending on panel’s decision. It is acknowledged that this approach has some limitations (i.e. panellists could not re-score the items considering the scores of other members, and it was not possible to compare agreement pre- and post-discussion); however, it was deemed to be the most appropriate approach as it reduced burden on panellists and the risk of attrition [3]. Furthermore, panellists were able to provide further feedback after having considered other members’ view during the online meeting and in phases 5 and 6. Open-ended questions were analysed following the guidelines provided by Côté, Salmela [25]. All responses were associated with a code which reflected the topic under discussion. Subsequently, answers with the same code (i.e. addressing the same topic) were grouped and merged into questions which were included in successive phases.

To examine the risk of attrition bias, the average scores of each round were calculated for each panel member and plotted as described in the COMET handbook [3]. Where the results of members who did not participate in subsequent rounds were similar to those completing all the rounds, attrition bias was deemed unlikely to influence the results. Following the Supreme Court model proposed by Shrier [26], the proportion of panellists who agreed and disagreed during each phase and the disagreement expressed during the online meeting were summarised and reported. Furthermore, the system was uploaded in a preprint to provide a communication channel to those not involved in the development of the system.

## Results

### Panellists

Fifteen of the 21 experts invited agreed to be included in the panel. From the 15 experts who agreed to participate, 12 completed the first phase (women *n* = 4; men *n* = 8). Eleven panellists were working in football in different leagues or national teams: English Premier League (*n* = 2), English Women’s Super League (*n* = 1), Qatar Stars League (*n* = 1), Spanish Primera Iberdrola (*n* = 1), Austrian BundesLiga (*n* = 1), Spanish La Liga (*n* = 1), European National Team (*n* = 1), Asian National Team (*n* = 2), South American National Team (*n* = 1). One panellist was working as a researcher only. The panellists had nine different nationalities: Australian (*n* = 3), British (*n* = 2), Brazilian (*n* = 1), Danish (*n* = 1), Dutch/Brazilian (*n* = 1), French (*n* = 1), Italian (*n* = 1), Japanese (*n* = 1), Spanish (*n* = 1). One panellist held a bachelor’s degree, two panellists held a master’s degree and nine panellists held a PhD. Panellists had experience working as an injury researcher (*n* = 3) or in professional football as a doctor (*n* = 4), physiotherapist (*n* = 5), S&C coach (*n* = 2) and sport scientist (*n* = 3). One panellist and a member of the steering committee have experience and education in the area of epidemiology. On average, panellists conducted research on injuries for 9.4 years (range 7–15 years) and worked in professional football for 12.6 years (range 7–30 years) (Fig. S1). All the duties included in the inclusion criteria described in Table 1 were fulfilled by at least four panellists (Fig. S2).

### Phase 1: Idea Generation

A list of the activities which lead to injury as reported in the literature was obtained from the studies included in the systematic review, and grouped into six domains: contact type, physical activity, ball situation, playing position, session detail and contextual information. Each domain was structured to include all activities reported in the literature. Therefore, the domains of the FIICCS were split into two sections: core set and optional set, which are separated by the horizontal lines in Fig. S3.

### Phase 2: First Ranking

In total, 12 panellists were asked to express their agreement through 49 unique questions (Table S1) with a total of 588 questions. In all questions, at least 60% of responders gave a response of 4 or 5. For 48 questions (98%) consensus was reached, while for one question (2%), relating to optional set on the ball situation, no consensus was reached (Figs. S4–S8), (Tables S2–S6).

Finally, the changes proposed by the panellists in the open-ended questions were collated into 30 questions (Table S7), which were included into the pre-meeting survey.

### Phase 3: Pre-meeting Survey

Panellists were asked to rate their agreement on the improvements suggested during the first survey. Of the 12 panellists who completed the first survey, 11 answered the pre-meeting survey while one panellist (doctor *n* = 1) could not continue the study due to limited availability.

**Fig. 1:**
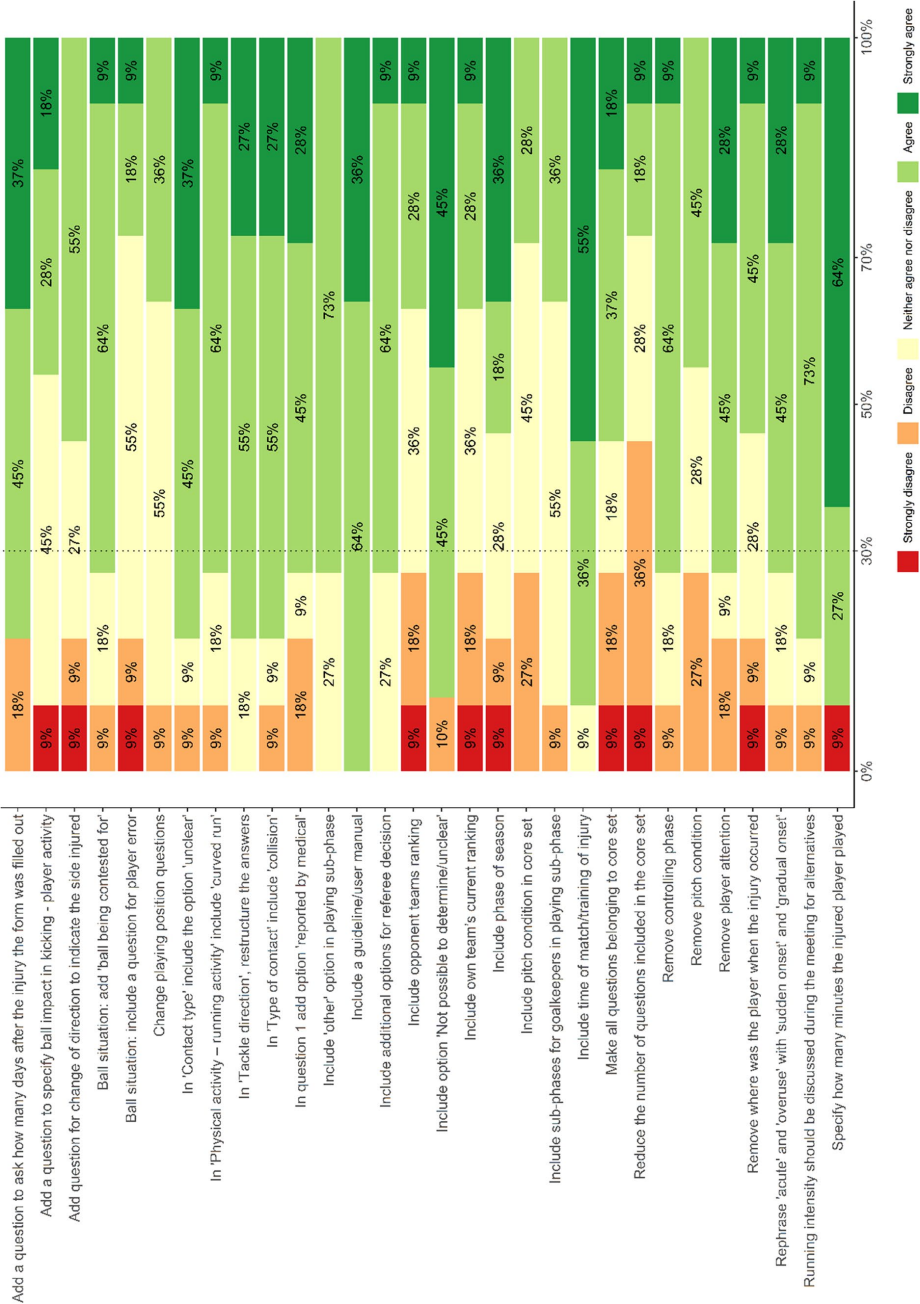
Agreement with changes proposed during the first survey expressed as a percentage of total answers

In total 11 panellists expressed their agreement through 30 questions (Table S7). Consensus was reached on 13 items, and the remaining 17 items were included in discussion (Fig. 1).

### Phase 4: Panel Discussion

Nine panellists participated in the online meeting, while two (S&C coach *n* = 1, doctor *n* = 1) could not attend it due to last-minute commitments with their football teams. One panellist (doctor) agreed to answer the survey, while the other (S&C coach) did not and was therefore excluded from further phases.

**Fig. 2:**
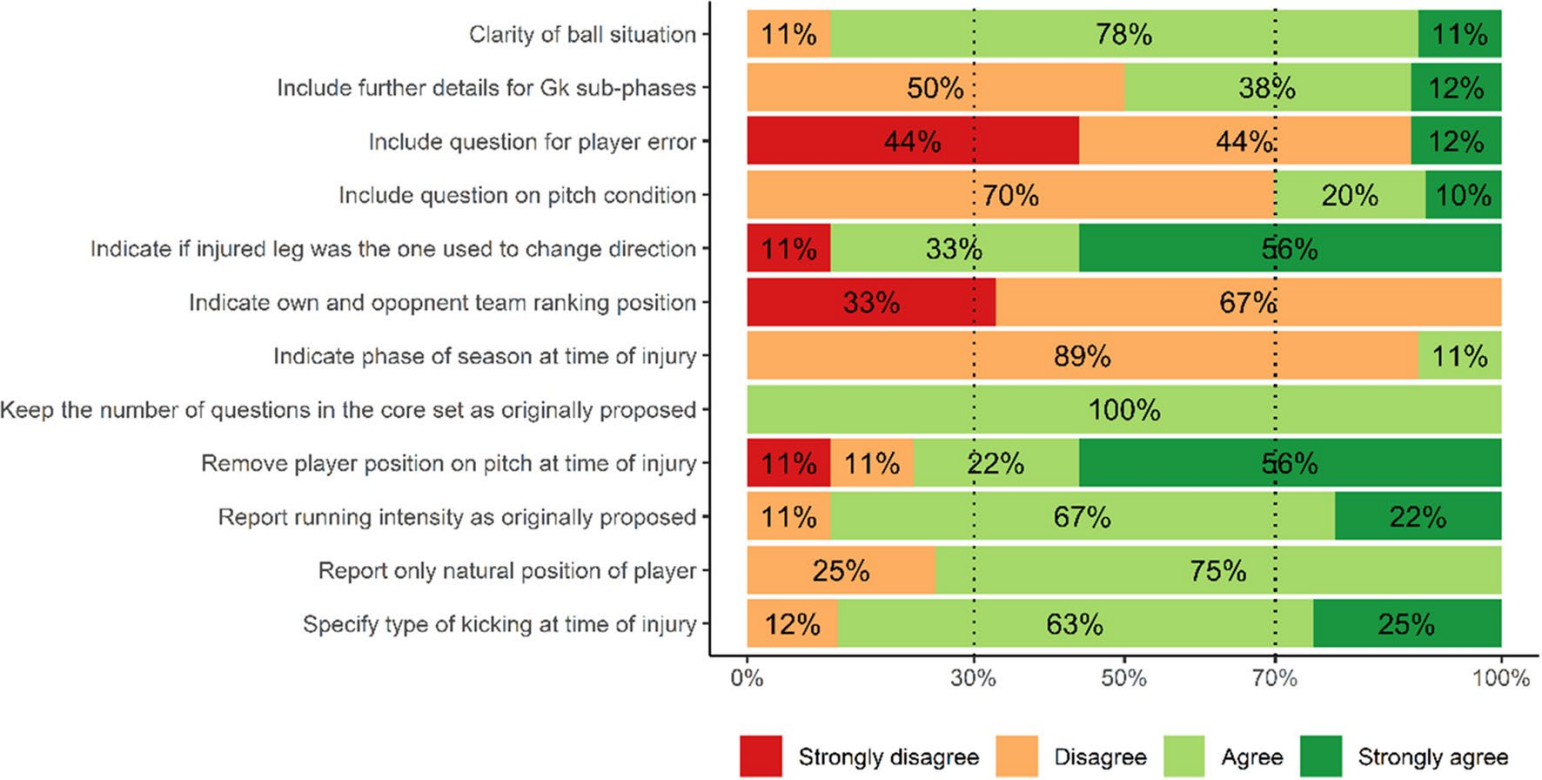
Panel responses on agreement after online discussion, expressed as a percentage of total number of answers

The meeting lasted 90 min, and all questions on which consensus was not achieved in the previous phases were discussed. Consensus was reached on 11 out of the 12 questions and was not achieved around whether further details in the description of goalkeeper-specific activities should be included (Fig. 2). When discussing the length of the core set, the panel appreciated that time demand is a concern when collecting information in the practical setting but argued that the actual length of the core set does not pose an excessive burden on practitioners and that a shorter core set would not allow the collection of sufficient information. One panellist suggested extending the length of the core set, but the panel finally agreed to keep the length of the core set as originally proposed. Following the changes proposed in phase 2 and accepted in phase 3, the panel deemed the optional set of ball situation section to be clear.

With reference to the definition of running intensity, the panel agreed that this is an important aspect to evaluate but that it must be acknowledged that not all teams can collect this information objectively. To do so, it would be necessary to monitor player activities through instruments such as global positioning systems (GPS) or optical video-tracking systems, and when an injury occurs, the inciting circumstance needs to be localised within the tracking data. Therefore, the panel agreed that, although the classification of running intensity originally proposed is not ideal, it seems the best way to collect such information as it allows the reporting of running intensity either using objective instruments or by using the reported categories (i.e. high intensity, medium intensity, low intensity) which are explained to standardise their interpretation. The panel agreed that if an acceptable inter-rater reliability of results will be confirmed in further studies, then this classification is reasonable; otherwise, alternatives or modifications will need to be considered.

With reference to including a question to indicate if the injured leg was the one used to perform the change of direction, it was discussed that this information could be useful especially for ankle, hip/groin and ligament injuries. With respect to including a question to indicate whether the injury occurred following player error, some panellists argued that it may be relevant because a player error (e.g. bad touch, wrong pass, wrong tactical behaviour) may lead the player to perform sudden and unusual movements, while others argued that this information may not be relevant because it cannot be used to develop prevention strategies. There was majority consensus that determining whether a player made an error is very subjective and there could be several different opinions of the same injury even when coaches are included in the decision; therefore, the panel agreed not to add this aspect into the FIICCS.

With reference to whether including a description on the type of kick performed at the time of injury was important, the panel agreed that this information is easy to collect and is relevant because each type of kick (e.g. inside kick, outside kick, back heel) involves different movements. This information could be used both to understand the mechanism of injury and to inform the development of rehabilitation protocols. With regard to whether pitch condition should be reported, some panellists argued that this information is important because pitch quality may vary significatively even in professional settings and may influence player load and the risk of some injuries such as ankle and knee injuries. On the other hand, other panellists argued that the quality of the pitch may not be homogeneous (i.e. some area of the pitch may be of good quality, and other areas may be of bad quality) and that reporting it may not be relevant because this information cannot be used for the development of prevention strategies. Regardless of the importance of reporting this information, there was consensus that evaluating pitch condition can be difficult because it is subjective and can change in different areas of the pitch; therefore, the panel agreed to exclude this question from the FIICCS.

With reference to player location on the pitch at the time of injury during match play, the panel agreed to remove this question because it is not important and does not provide important information for the development of prevention strategies. With respect to reporting playing position, it was argued that people reporting inciting circumstances may not know the position the player was playing in during at the time of injury because players change position very frequently during football games; therefore, it is difficult to collect and report the information accurately. Therefore, the panel agreed to report only the natural playing position (i.e. the position in which the player usually plays in). The panel agreed not to collect information on teams ranking position or phase of season at the time of injury because it was deemed not worth collecting through this process and because the information can be extrapolated from the date of injury as required.

Finally, the panel discussed whether more information on the goalkeeper-specific activity should be reported. It was discussed that the FIICCS allows the reporting of the most important inciting activities (e.g. landing, kicking, diving); therefore, it may not be relevant to include further information. However, consensus was not reached because only 50% of the panellists agreed not to include further information. Therefore, since the reporting of goalkeeper-specific inciting activities is included in the optional set, no further information was included in the sub-domain, but further details can be collected if the researchers or practitioners deem this to be necessary.

### Phase 5 and 6: Alignment with FIFA Football Language and Football Injury Surveillance Methodology Consensus

The FIICCS was updated following panel comments, the publication of the FIFA Football Language and the football extension of the International Olympic Committee consensus on methods for recording and reporting of epidemiological data on injury and illness in sport and was finally sent to the panellists for final approval. All the panellists confirmed their agreement with the final version and did not have any further comment.

### Final Classification System

**Fig. 3:**
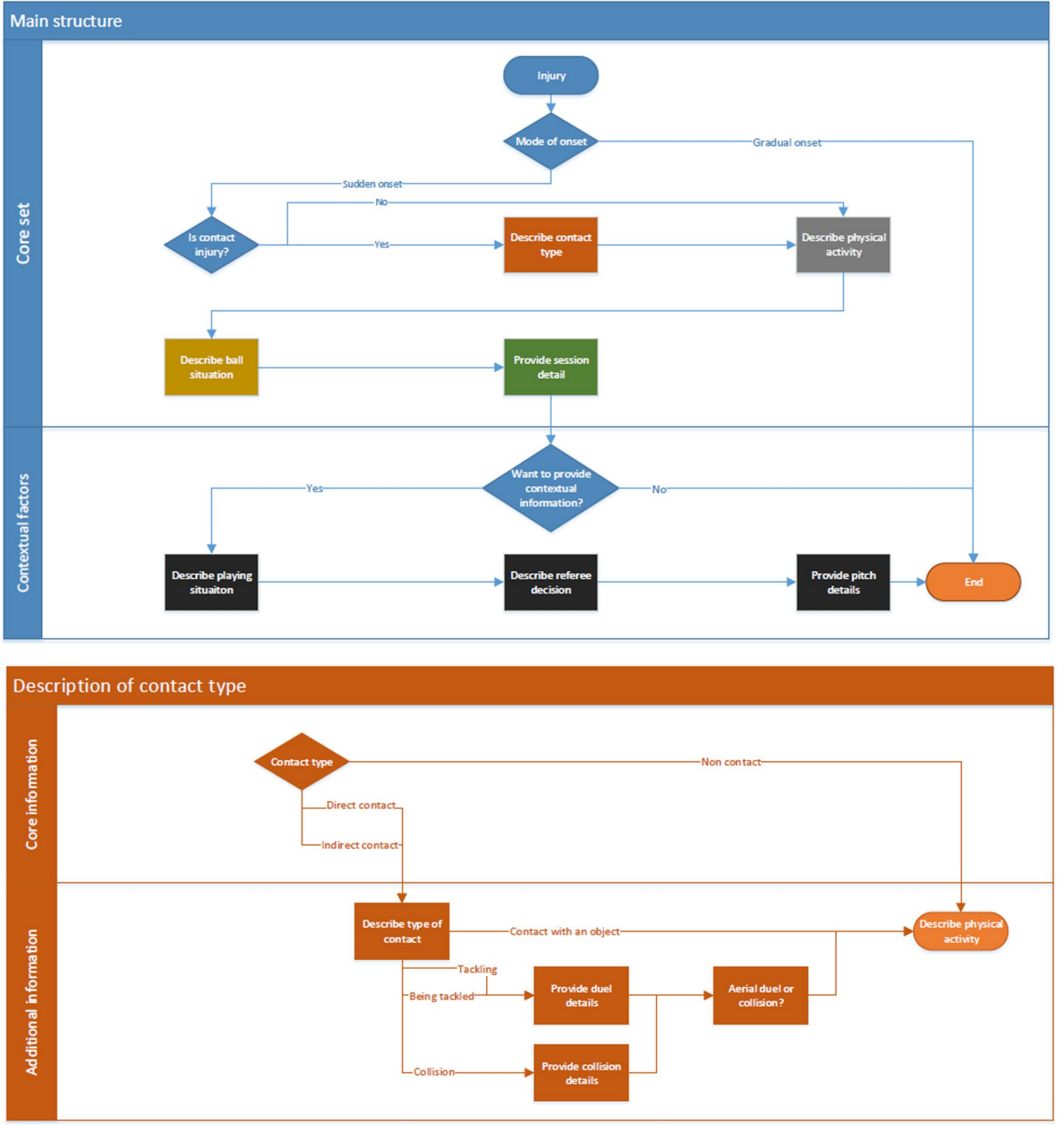

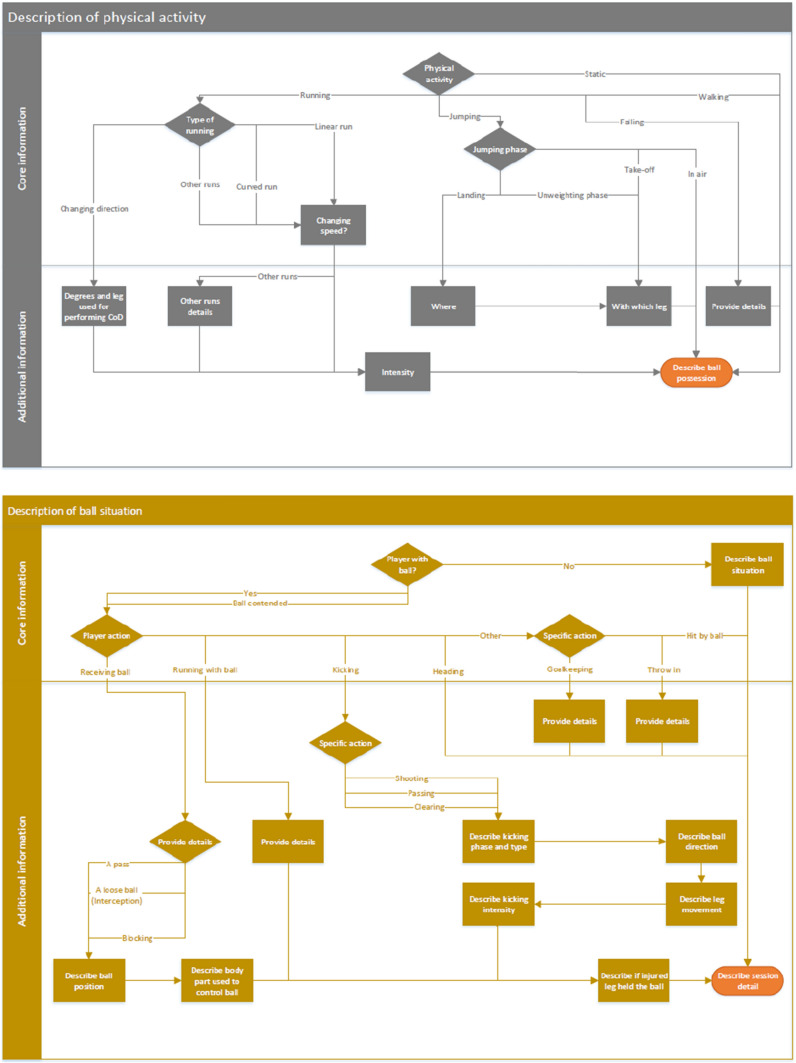

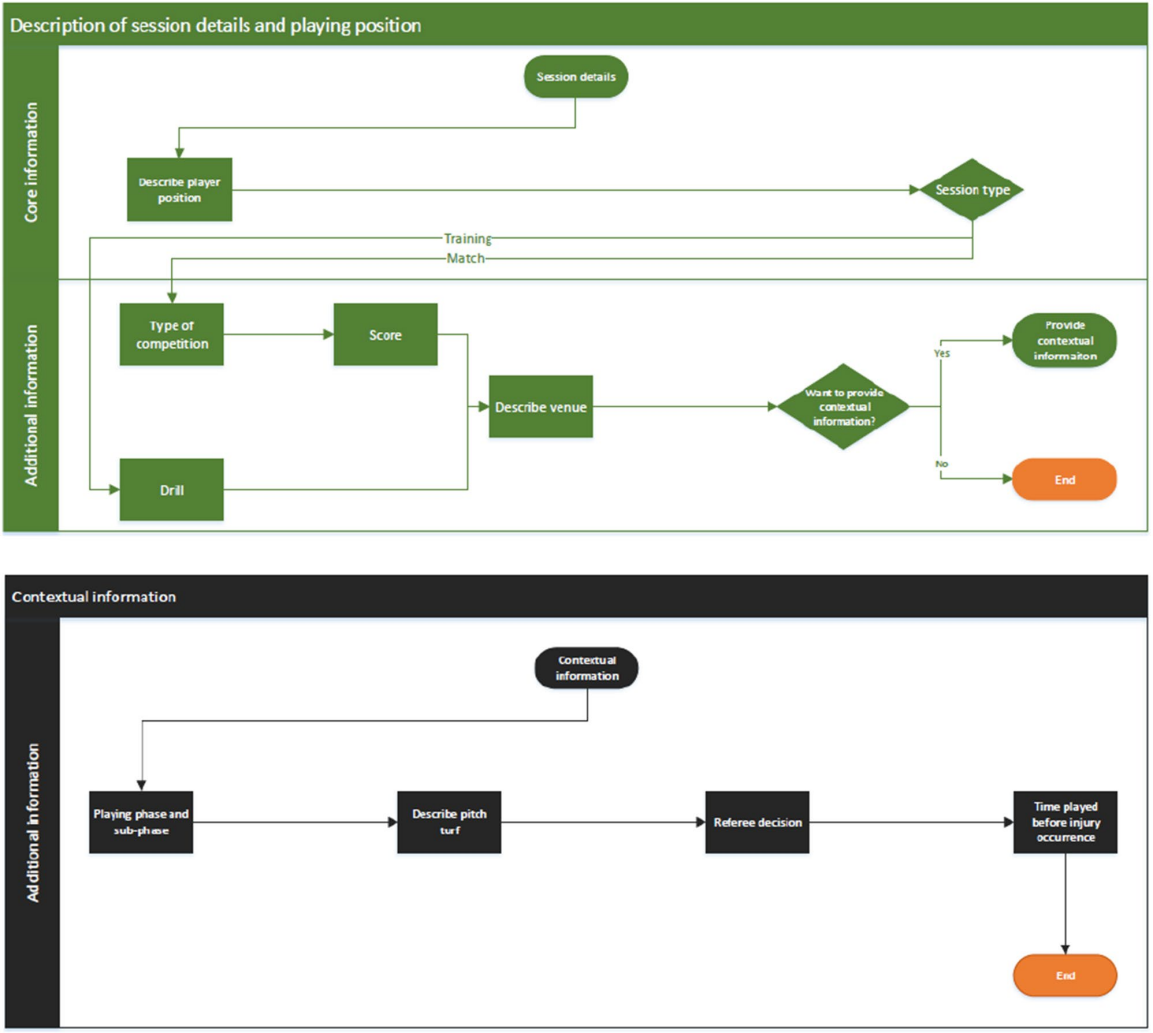
Final structure of the injury FIICCS. The flow chart should be read from the top to the bottom and from left to right following the arrows. The first figure (main structure) describes the structure of the FIICCS and its elements. The following figures (e.g. description of contact type, description of physical activity) describe in detail each element of the FIICCS. The description of each element has been reported in the excel file available at https://osf.io/3h59v

The final version of the FIICCS is available in Excel format (https://osf.io/3h59v), and its structure is described in Fig. 3. The user guidelines with the definition of the domains and sub-domains of the core and optional sets are available at the same link. They have been developed by the Steering Committee, revised by the panellists, further revised by the steering committee and one panellist as part of the development of the medical coding of the FIFA Football Language, and finally approved by the panel.

### Attrition

Twelve panellists originally agreed to participate in the study and completed the second phase. One panellist (doctor) left the study after the second phase, and one panellist (S&C coach) left the study after the third phase. Both panellists declared that they could not continue the study due to their limited availability to be able to meet the necessary time commitment. To evaluate whether attrition in phase 3 and 4 introduced bias, the average score of the answers provided by each panellist who left the study was compared with the average score of the answers provided by the panellists who completed all the phases as per COMET guidelines [3]. For example, the average score of the nine questions related to importance was calculated for each panellist, and then the individual averages were compared to evaluate whether the ones of the panellists who dropped out differed from the ones of the panellists who completed the study. The average score of those who did not complete phase 3 or phase 4 were within the range of the average score of the panellists who completed the study (Fig. S9), which suggests that attrition did not introduce bias.

## Discussion

Following robust consensus methods and the COMET guidelines, a standardised classification system was developed for use by practitioners and researchers to systematically report the inciting circumstances in football. The FIICCS comprises five domains: contact type, running activity, ball situation, session details and contextual information. The system also distinguishes between core set (essential reporting) and optional set (additional details). The core set allows the reporting of information deemed necessary at a minimum level to evaluate the inciting circumstances and means that this information should always be reported. It has been deemed to be easy to use and not excessively time demanding by the panellists. The optional set is longer and allows the reporting of more in-depth detail on the inciting circumstances. It has been deemed to be easy to use in practical and research settings, but to report the information requested it is required to view the video clip of the inciting circumstances. The optional set can be also adapted by the researchers or practitioners who wish to collect different or additional information on the inciting circumstances and depends on the research question or the question posed in the practical setting. The core set, which on the contrary should not be modified, will encourage sufficient reporting consistency and addresses the issues of inconsistent reporting observed within the literature.

The FIICCS can be used as a tool to collect and report inciting circumstances, but we recommend using it with caution until the system is evaluated further for reliability of reporting. Practitioners and researchers working with football clubs may easily include the core set in the report forms they routinely use to collect injury data. This increases the chances of the core set being implemented because it is not time demanding and can be included into injury monitoring procedures already in place. The optional set has been deemed easy to implement in football environments by the panel; however, since it is more time demanding, its implementation may encounter some barriers. On the other hand, both the core set and the optional set can be easily implemented within research settings. Indeed, researchers could be interested in performing detailed analyses of the inciting circumstances and in some cases could decide to change the optional set according to their purposes. For example, if the study aims to evaluate the biomechanics of anterior cruciate ligament injuries or to report in detail the tactical circumstances at the time of injury, appropriate optional sets would need to be implemented. It is recognised that following its implementation it is possible that further improvements will arise and the system will need to be updated, but this is a normal part of the scientific process [27, 28]. Additionally, it will be necessary to evaluate the reliability of the system, which might be reduced in some situations such as when evaluating running intensity without appropriate instrumentation. Given the extent of reporting inconsistency of inciting circumstances in the available literature [2], the system could be implemented to reduce this issue while further studies evaluate its reliability. We urge future studies to report the inciting circumstances following the FIICCS. This will allow researchers and practitioners to combine, compare and generalise findings across studies and then to identify possible mechanisms of injury, inform practitioners and develop injury prevention strategies that can be tested.

Despite following the COMET guidelines, this study is not without limitations. The study protocol was not registered, and our original protocol had to be modified to meet the limited availability of the panellists, whose involvement was crucial for the development of the FIICCS. Furthermore, there is significant uncertainty on methods to develop core outcome set [3]. As a consequence, some decisions (e.g. the cut-off value to deem consensus reached, the exclusion of the neutral answer in phase 4) were taken on the basis of the experience of the steering committee and following the methodology implemented in similar studies. Further limitations concern how the panel was recruited and the risk of attrition bias. Recruiting panellists from the network of the steering committee might have led the panel to be formed by experts whose opinions are similar to those of the steering committees. Using purposive sampling was necessary due to the difficulties in recruiting practitioners working in professional football as reported in other consensus studies [21, 22]. Indeed, only 12 out of 21 experts agreed to participate, while others either did not answer the invitation or refused due to time constraints. The number of answers not provided in the surveys might be an additional limitation. However, it is believed that panellists did not provide answers when they did not have a clear opinion or due to lack of time. In any case, to limit the potential impact of the answers not provided on the FIICCS, panellists were requested to review and confirm their agreement with the FIICCS twice (phases 5 and 6).

Furthermore, despite every effort being put in place to reduce attrition, 2/12 (17%) of panellists did not complete the study. The risk of attrition bias was low, but the opinions of the panellists who did not complete the study might have led to different decisions. Another limitation is that panellists had to be fluent English speakers, which reduces the representativeness of those who do not speak English. Additionally, since panellists were researchers and/or practitioners working at professional level, it is unclear whether the FIICCS is applicable at lower levels where the medical and support staff availability is limited. Direct applicability or adjustments needed will require further investigation in due time. Finally, we acknowledge that classifying injuries as contact and non-contact as per STROBE-SIIS guidelines [7] may be challenging as suggested by Shrier [26] and that this will deserve further consideration which was beyond the scope of this study.

## Conclusions

We have developed a standardised system that allows practitioners and researchers to systematically report inciting circumstances leading to injury in football. This comprises a core outcome set and an optional set. The core set is short, can be easily used in research and/or practice environment, and can be included in the injury reporting routines already in place in football, while the optional set can be implemented in both football and research environments but could require video analysis. Following recommendations from Shrier [26], we reported in detail the disagreements that arose within the panel and uploaded the FIICCS in excel format in a preprint (https://osf.io/3h59v) to provide a communication channel to those not involved in the development, and we invite those not involved in the development to use the FIICCS and this platform to provide feedback.

## Supplementary Information

Below is the link to the electronic supplementary material.

Supplementary file1 (PDF 892 KB)

## Declarations

### Availability of data and material

All data generated or analysed during this study are included in this published article (and its supplementary information files).

## References

[1] O’Brien J, Finch CF, Pruna R, McCall A (2019). A new model for injury prevention in team sports: the Team-sport Injury Prevention (TIP) cycle. Sci Med Football..

[2] Aiello F, Impellizzeri FM, Brown SJ, Serner A, McCall A (2022). Injury-inciting activities in male and female football players: a systematic review. Sports Med.

[3] Williamson PR, Altman DG, Bagley H, Barnes KL, Blazeby JM, Brookes ST (2017). The COMET handbook: version 1.0. Trials.

[4] Clarke M, Williamson PR (2016). Core outcome sets and systematic reviews. Syst Rev..

[5] Clarke M (2007). Standardising outcomes for clinical trials and systematic reviews. Trials.

[6] Hutton JL, Williamson PR (2000). Bias in meta-analysis due to outcome variable selection within studies. J Roy Stat Soc Ser C (Appl Stat).

[7] Bahr R, Clarsen B, Derman W, Dvorak J, Emery CA, Finch CF (2020). International Olympic Committee consensus statement: methods for recording and reporting of epidemiological data on injury and illness in sport 2020 (including STROBE Extension for Sport Injury and Illness Surveillance (STROBE-SIIS)). Br J Sports Med.

[8] Andersen TE, Larsen O, Tenga A, Engebretsen L, Bahr R (2003). Football incident analysis: a new video based method to describe injury mechanisms in professional football. Br J Sports Med.

[9] Black N. Consensus development methods. Qualitative research in health care, 3rd edn. Hoboken, New Jersey: Wiley; 2006.

[10] Black N, Murphy M, Lamping D, McKee M, Sanderson C, Askham J (1999). Consensus development methods: a review of best practice in creating clinical guidelines. J Health Serv Res Policy.

[11] McMillan SS, King M, Tully MP (2016). How to use the nominal group and Delphi techniques. Int J Clin Pharm.

[12] Richardson FM (1972). Peer review of medical care. Med Care.

[13] FIFA. FIFA Football Language. 2022 [cited 12/01/2022]. https://www.fifatrainingcentre.com/en/resources-tools/football-language/. Accessed 12 Jan 2022.

[14] Waldén M, Mountjoy M, McCall A, Serner A, Massey A, Tol JL, Bahr R, D'Hooghe M, Bittencourt N, Della Villa F, Dohi M, Dupont G, Fulcher M, van Rensburg DCJ, Lu D, Andersen TE (2023) Football-specific extension of the IOC consensus statement: methods for recording and reporting of epidemiological data on injury and illness in sport 2020. Br J Sports Med. 10.1136/bjsports-2022-106405 10.1136/bjsports-2022-106405 PMC10646851 36609352

[15] Hasson F, Keeney S, McKenna H (2000). Research guidelines for the Delphi survey technique. J Adv Nurs.

[16] Vagias WM. Likert-type scale response anchors. Clemson International Institute for Tourism & Research Development, Department of Parks, Recreation and Tourism Management. Clemson University. 2006. http://media.clemson.edu/cbshs/prtm/research/resources-for-research-page-2/Vagias-Likert-Type-Scale-Response-Anchors.pdf.

[17] Eklund A, Trimble J. beeswarm: The Bee Swarm Plot, an Alternative to Stripchart. R package version 0.4.0 ed; 2021.

[18] Wickham H (2016). ggplot2: Elegant graphics for data analysis.

[19] RStudio Team. RStudio: integrated development for R. RStudio, PBC.; 2020.

[20] Wilke C. cowplot: Streamlined plot theme and plot annotations for ‘ggplot2’. 2020.

[21] McCall A, Pruna R, Van der Horst N, Dupont G, Buchheit M, Coutts AJ (2020). Exercise-based strategies to prevent muscle injury in male elite footballers: an expert-led Delphi survey of 21 practitioners belonging to 18 teams from the Big-5 European Leagues. Sports Med.

[22] Zambaldi M, Beasley I, Rushton A (2017). Return to play criteria after hamstring muscle injury in professional football: a Delphi consensus study. Br J Sports Med.

[23] van der Horst N, Backx F, Goedhart EA, Huisstede BM (2017). Return to play after hamstring injuries in football (soccer): a worldwide Delphi procedure regarding definition, medical criteria and decision-making. Br J Sports Med.

[24] McPherson S, Reese C, Wendler MC (2018). Methodology update: Delphi studies. Nurs Res..

[25] Côté J, Salmela JH, Baria A, Russell SJ (1993). Organizing and interpreting unstructured qualitative data. Sport Psychol.

[26] Shrier I (2021). Consensus statements that fail to recognise dissent are flawed by design: a narrative review with 10 suggested improvements. Br J Sports Med.

[27] Finch CF, Cook J (2014). Categorising sports injuries in epidemiological studies: the subsequent injury categorisation (SIC) model to address multiple, recurrent and exacerbation of injuries. Br J Sports Med.

[28] Clarsen B, Bahr R, Myklebust G, Andersson SH, Docking SI, Drew M (2020). Improved reporting of overuse injuries and health problems in sport: an update of the Oslo Sport Trauma Research Center questionnaires. Br J Sports Med.

