# Supplementary material for: Development of a Standardised System to Classify Injury-Inciting Circumstances in Football: the Football Injury Inciting Circumstances Classification System (FIICCS)
Source: Sports Med. 2023 May 26;53(9):1805–18. doi: 10.1007/s40279-023-01857-6 (PMC10432371; doi:10.1007/s40279-023-01857-6)
Supplement: Supplementary file 1 — Supplementary file1 (PDF 892 KB) [file 40279_2023_1857_MOESM1_ESM.pdf]

Development of a standardised system to classify injury inciting circumstances in football: the Football Injury Inciting Circumstances Classification System (FIICCS)

Francesco Aiello[1,2], Alan McCall[1,2*], Susan J. Brown[2], Andreas Serner[3], Lauren V. Fortington[4], Suzanne Afra Elisabeth Huurman[5,6], Colin Lewin[7], Masashi Nagao[8,9,10], James O'Brien[11], Anastasia Panossian[12], Ricard Pruna[13], Guilherme Passos Ramos[14,15], Matthew Whalan[16,17], Franco M. Impellizzeri[18]

1 Arsenal Performance and Research Team, Arsenal Football Club, London, UK

2 School of Applied Sciences, Edinburgh Napier University, Edinburgh, UK

3 Fédération Internationale de Football Association, Zurich, Switzerland

4 School of Medical and Health Sciences, Edith Cowan University, Australia

5 Medical Department Real Madrid CF, Madrid, Spain

6 Sports Medicine Department St Antonius Hospital, Utrecht, the Netherlands

7 The Lewin Sports Injury Clinic, East London, UK

8 Medical Technology Innovation Center, Juntendo University, Bunkyo-Kku, Tokyo, Japan

9 Department of Orthopaedic Surgery, Juntendo University Faculty of Medicine, Bunkyo-Kku, Tokyo, Japan

10 Department of Sports Medicine, Juntendo University, Bunkyo-Ku, Tokyo, Japan

11 Red Bull Athlete Performance Center, Salzburg, Austria

12 Chelsea FC, London, UK

13 FC Barcelona Medical Services, Barcelona, Spain

14 Brazilian Football Confederation (CBF), Rio de Janeiro, RJ, Brazil.

15 Endocrinology and Metabolism Laboratory, Department of Physiology and Biophysics, Federal University of Minas Gerais (UFMG), Belo Horizonte, MG, Brazil.

16 Centre of Medical and Exercise Physiology, School of Medicine, University of Wollongong, Wollongong, New South Wales, Australia

17 Football Australia, Sydney, Australia

18 Faculty of Health, Sport and Exercise Discipline Group, University of Technology Sydney, Sydney, Australia

*Corresponding author: Alan McCall, a.mccall@napier.ac.uk

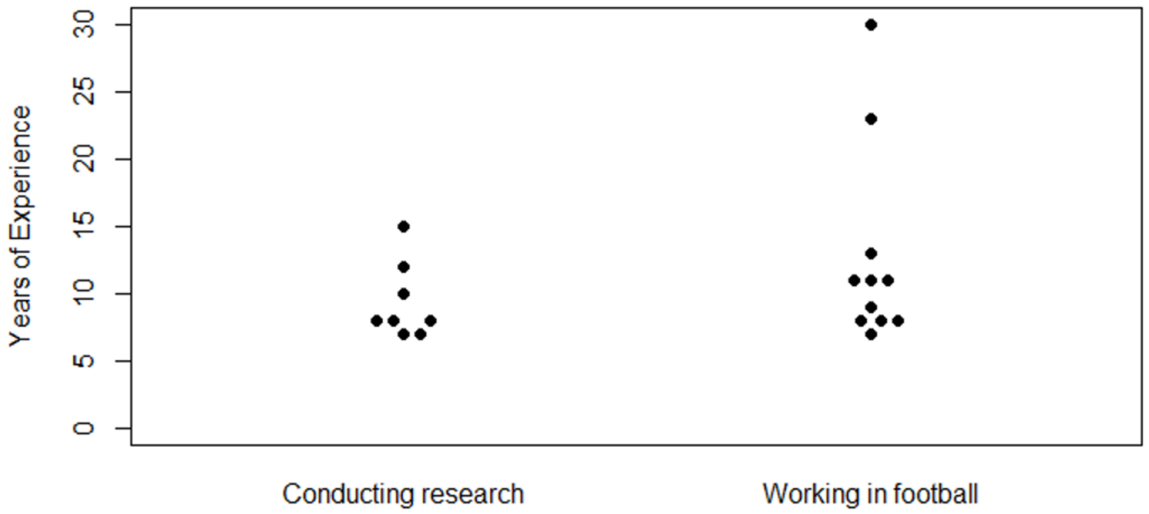

Fig. S1 Panellists' experience (years)

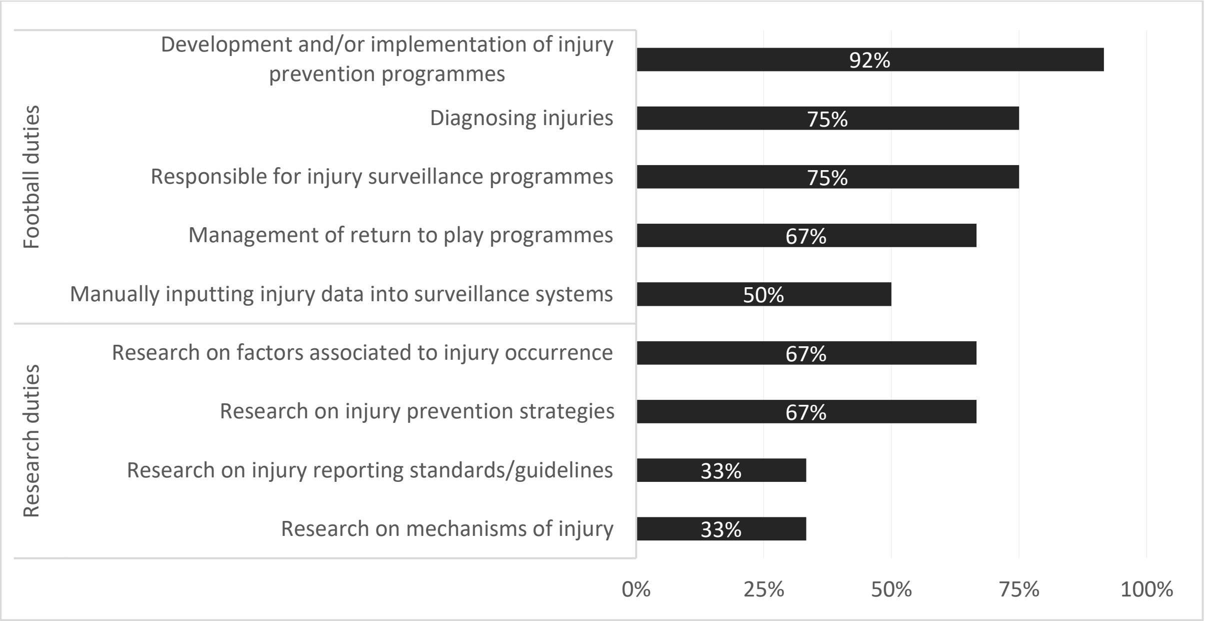

Fig. S2 Panellists' duties (expressed as a percentage of all panellists)

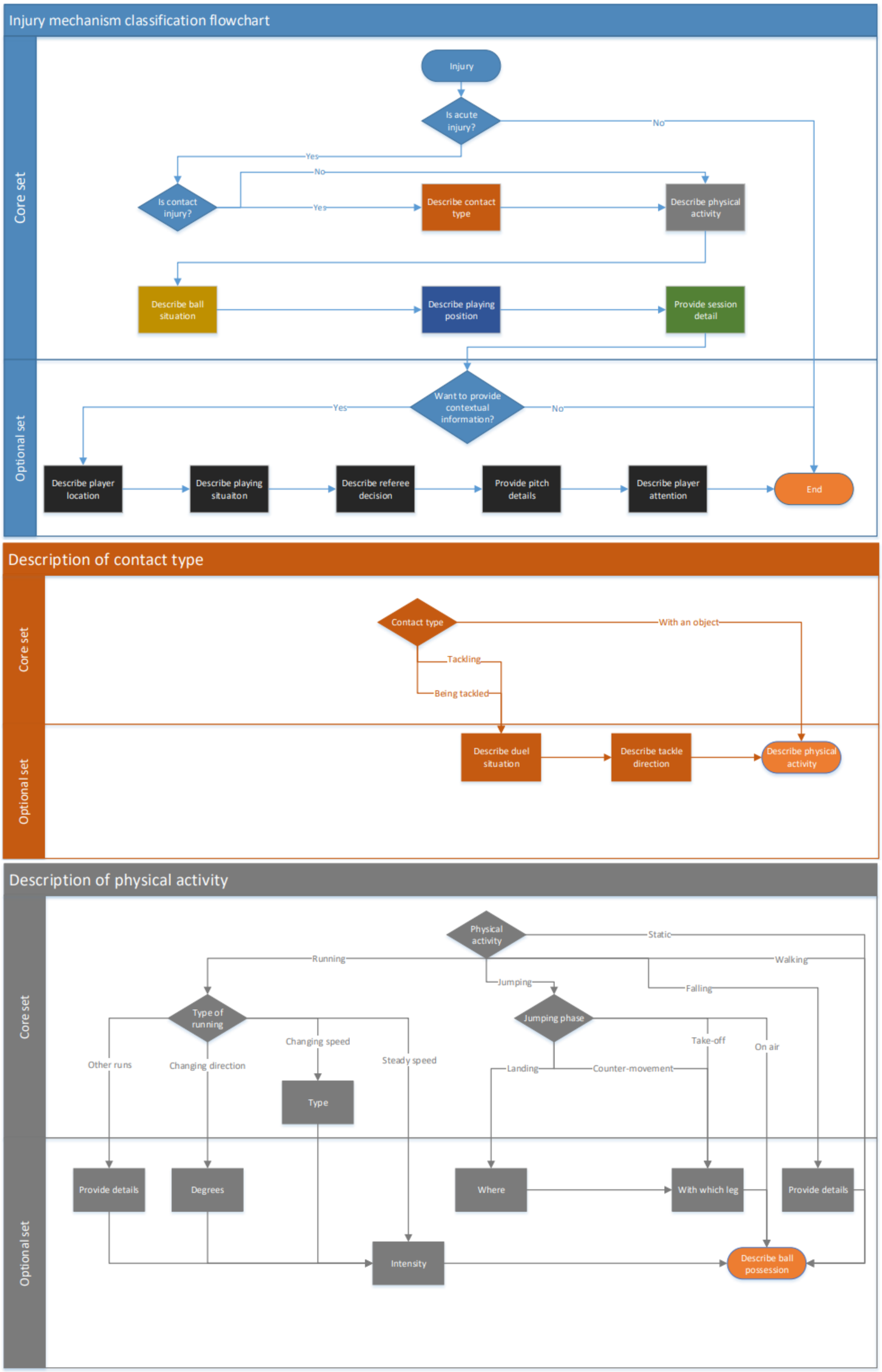
Injury mechanism classification flowchart
Core set
Optional set
Injury
Is acute injury?
No
Yes
No
Is contact injury?
Yes
Describe contact type
Describe physical activity
Describe ball situation
Describe playing position
Provide session detail
Want to provide contextual information?
Yes
No
Describe player location
Describe playing situaiton
Describe referee decision
Provide pitch details
Describe player attention
End
Description of contact type
Core set
Optional set
Contact type
With an object
Tackling
Being tackled
Describe duel situation
Describe tackle direction
Describe physical activity
Description of physical activity
Core set
Optional set
Physical activity
Static
Running
Walking
Jumping
Falling
Type of running
Jumping phase
Changing speed
Take-off
On air
Other runs
Changing direction
Steady speed
Landing
Counter-movement
Type
Provide details
Degrees
Where
With which leg
Provide details
Intensity
Describe ball possession

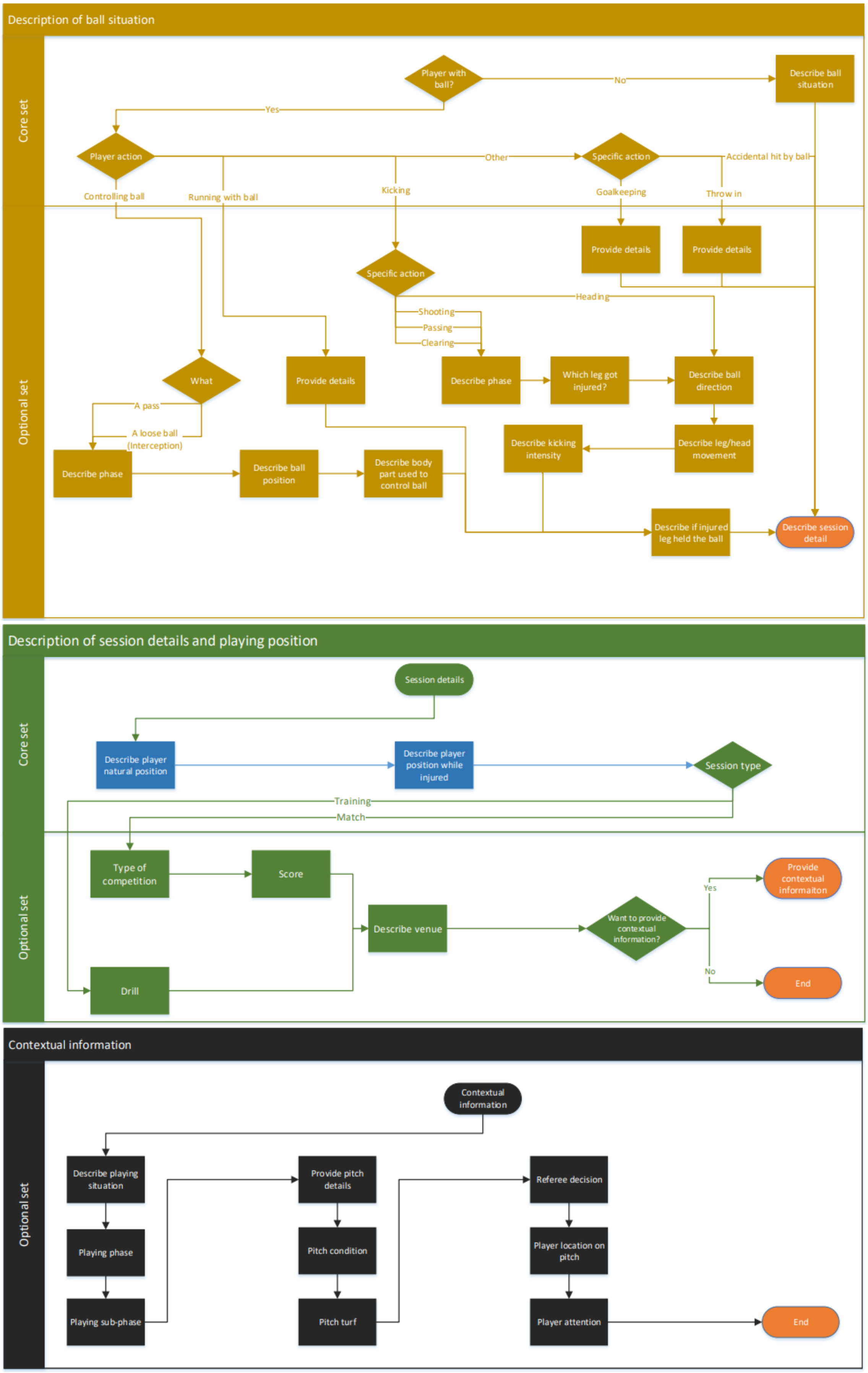

Fig. S3 Draft of the structure of the injury FIICCS used in phase 2

Table S1 Questions included in phase two survey

Question Number	Question	Responses
1	Please rate how much you agree with the content and the organisation of the following sections of the system:<br>1) Main structure - Core set;<br>2) Contact type - Core set;<br>3) Physical activity - Core set;<br>4) Ball situation - Core set;<br>5) Session detail - Core set;<br>6) Main structure - Optional set;<br>7) Contact type - Optional set<br>8) Physical activity - Optional set;<br>9) Ball situation - Optional set;<br>10) Session detail - Optional set;<br>11) Contextual information - Optional set	1 = Strongly disagree;<br>2 = Disagree;<br>3 = Neither agree nor disagree;<br>4 = Agree;<br>5 = Strongly agree;<br>NA = Prefer not to say
2	Do you have further feedback on the content and the organisation of the sections? Would you add/exclude some from the core set or the whole structure?	Open-ended question
3	Do you have any feedback on the items of each section (e.g., jumping, running, kicking)? Would you add/exclude some or change how they are structured within the sections?	Open-ended question

4	How important is including the following information when reporting injury mechanisms [i.e. inciting circumstances] in football?<br>1) Contact type - Core set;<br>2) Physical activity - Core set;<br>3) Ball situation - Core set;<br>4) Session detail - Core set;<br>5) Contact type - Optional set;<br>6) Physical activity - Optional set;<br>7) Ball situation - Optional set;<br>8) Session detail - Optional set;<br>9) Contextual information - Optional set	1 = Not at all;<br>2 = Slight;<br>3 = Neutral;<br>4 = Moderately;<br>5 = Extremely;<br>NA = Prefer not to say
5	Do you have further feedback on the importance of each section?	Open-ended question
6	Please rate the clarity of each section:<br>1) Main structure - Core set;<br>2) Contact type - Core set;<br>3) Physical activity - Core set;<br>4) Ball situation - Core set;<br>5) Session detail - Core set;<br>6) Main structure - Optional set;<br>7) Contact type - Optional set<br>8) Physical activity - Optional set;<br>9) Ball situation - Optional set;<br>10) Session detail - Optional set;	1 = Poor;<br>2 = Fair;<br>3 = Good;<br>4 = Very good;<br>5 = Excellent;<br>NA = Prefer not to say

	11) Contextual information - Optional set	
7	Which parts of the sections need to be revised to improve clarity?	Open-ended question
8	How difficult would it be to collect information on each section in football environment?<br>1) Contact type - Core set;<br>2) Physical activity - Core set;<br>3) Ball situation - Core set;<br>4) Session detail - Core set;<br>5) Contact type - Optional set;<br>6) Physical activity - Optional set;<br>7) Ball situation - Optional set;<br>8) Session detail - Optional set;<br>9) Contextual information - Optional set	1 = Very difficult;<br>2 = Difficult;<br>3 = Neutral;<br>4 = Easy;<br>5 = Very easy;<br>NA = Prefer not to say
9	Do you have further feedback on how the system could be improved to make it easier to implement in football environment?	Open-ended question
10	How difficult would implementing the system in research environment be?<br>1) Contact type - Core set;<br>2) Physical activity - Core set;<br>3) Ball situation - Core set;<br>4) Session detail - Core set;<br>5) Contact type - Optional set;<br>6) Physical activity - Optional set;	1 = Very difficult;<br>2 = Difficult;<br>3 = Neutral;<br>4 = Easy;<br>5 = Very easy;<br>NA = Prefer not to say

	7) Ball situation - Optional set;	
	8) Session detail - Optional set;	
	9) Contextual information - Optional set	
11	Do you have further feedback on how the system could be improved to make it easier to implement in research environment?	Open-ended question
12	Do you have further feedback or recommendation to improve the classification system?	Open-ended question

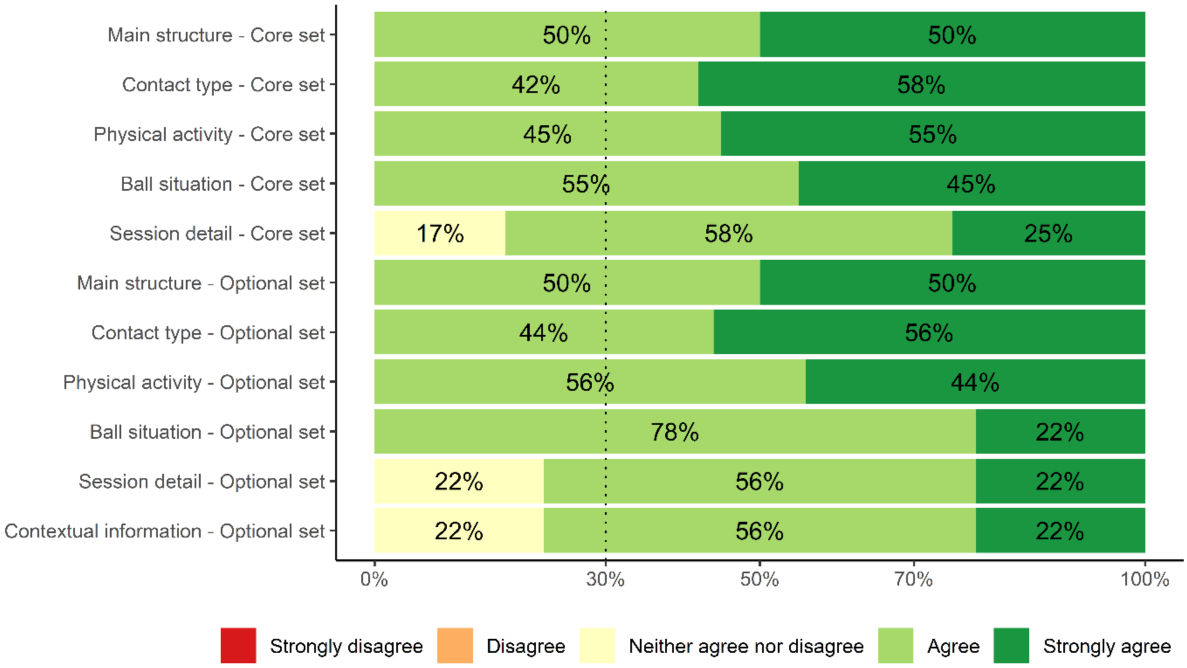

Fig. S4 Agreement with content and organisation of the domains

Table S2 Answers on the agreement with content and organisation of the domains

**Domain**	**1**	**2**	**3**	**4**	**5**	**Total**
Main structure - Core set	0	0	0	6	6	12
Contact type - Core set	0	0	0	5	7	12
Physical activity - Core set	0	0	0	5	6	11

Ball situation - Core set	0	0	0	6	5	11
Session detail - Core set	0	0	2	7	3	12
Main structure - Optional set	0	0	0	4	4	8
Contact type - Optional set	0	0	0	4	5	9
Physical activity - Optional set	0	0	0	5	4	9
Ball situation - Optional set	0	0	0	7	2	9
Session detail - Optional set	0	0	2	5	2	9
Contextual information - Optional set	0	0	2	5	2	9

1 = Strongly disagree; 2 = Disagree; 3 = Neither agree nor disagree; 4 = Agree; 5 = Strongly agree
Total number of panellists included in this round = 12. The difference between the total number of panellists and the total number of answers provided represents the number of answers not given for each question

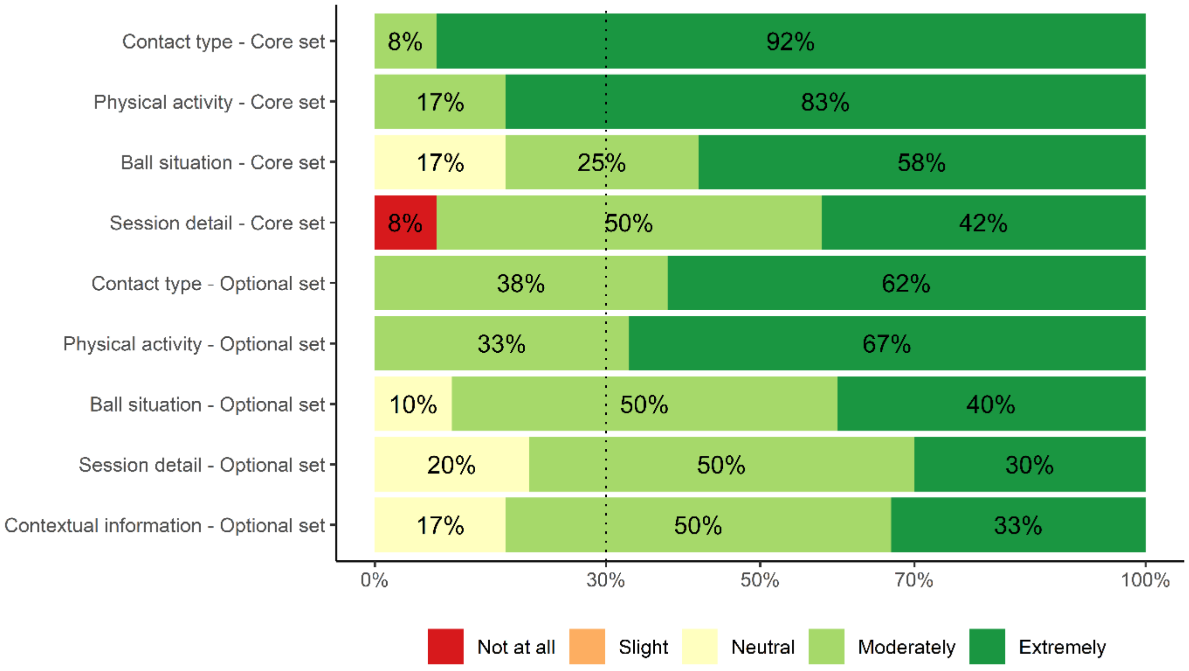

Fig. S5 Consensus around the importance of reporting the domains

Table S3 Answers on the Importance of reporting each domain

**Domain**	**1**	**2**	**3**	**4**	**5**	**Total**

Contact type - Core set	0	0	0	1	11	12
Physical activity - Core set	0	0	0	2	10	12
Ball situation - Core set	0	0	2	3	7	12
Session detail - Core set	1	0	0	6	5	12
Contact type - Optional set	0	0	0	3	5	8
Physical activity - Optional set	0	0	0	3	6	9
Ball situation - Optional set	0	0	1	5	4	10
Session detail - Optional set	0	0	2	5	3	10
Contextual information - Optional set	0	0	2	6	4	12

1 = Not at all; 2 = Slight; 3 = Neutral; 4 = Moderately; 5 = Extremely
Total number of panellists included in this round = 12. The difference between the total number of panellists and the total number of answers provided represents the number of answers not given for each question

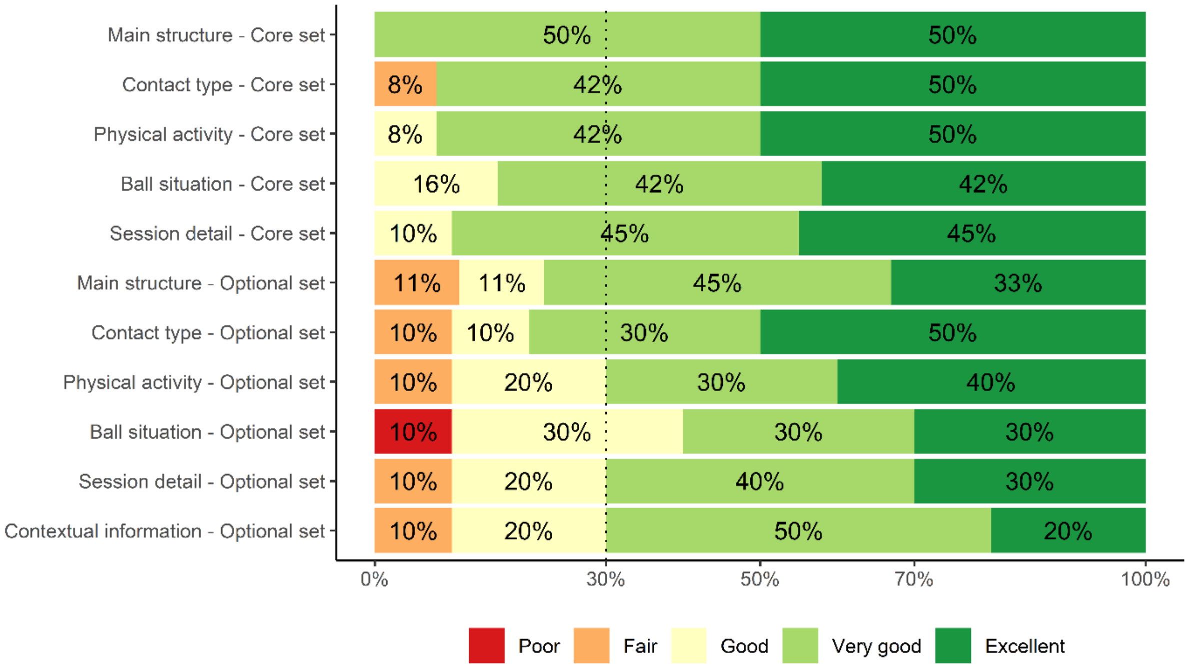

Fig. S6 Consensus around the clarity of the domains

Table S4 Answers on the clarity of the domains

**Domain**	**1**	**2**	**3**	**4**	**5**	**Total**
Main structure - Core set	0	0	0	6	6	12

Contact type - Core set	0	1	0	5	6	12
Physical activity - Core set	0	0	1	5	6	12
Ball situation - Core set	0	0	2	5	5	12
Session detail - Core set	0	0	1	5	5	11
Main structure - Optional set	0	1	1	4	3	9
Contact type - Optional set	0	1	1	3	5	10
Physical activity - Optional set	0	1	2	3	4	10
Ball situation - Optional set	1	0	3	3	3	10
Session detail - Optional set	0	1	2	4	3	10
Contextual information - Optional set	0	1	2	5	2	10

1 = Poor; 2 = Fair; 3 = Good; 4 = Very good; 5 = Excellent
Total number of panellists included in this round = 12. The difference between the total number of panellists and the total number of answers provided represents the number of answers not given for each question

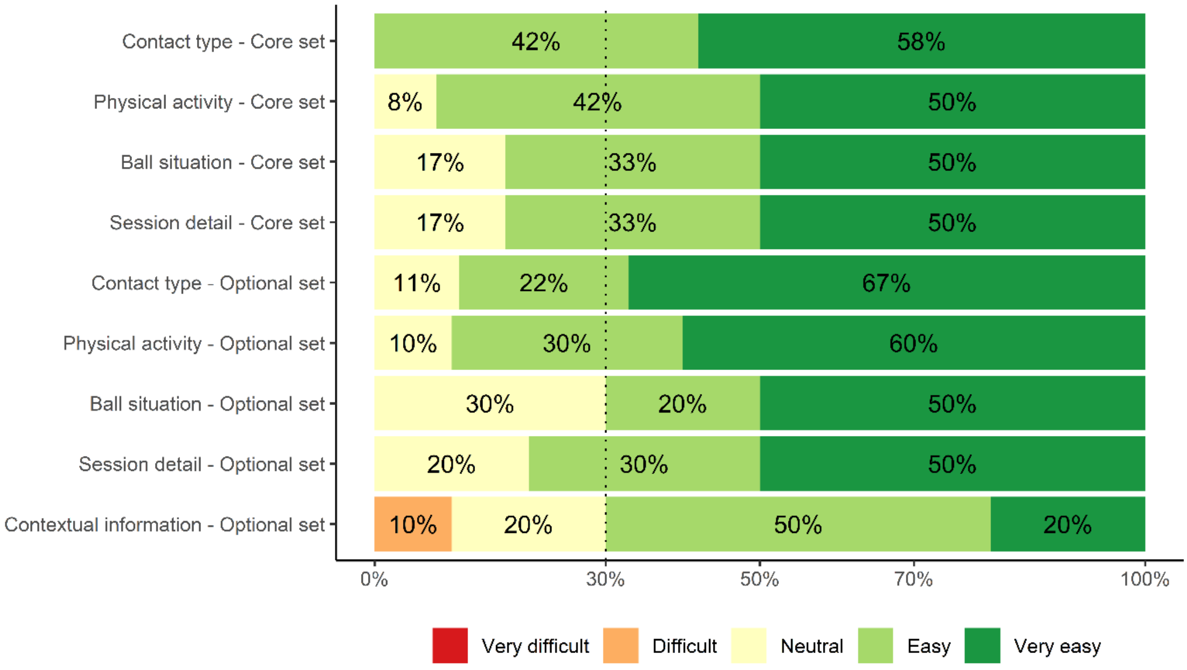

Fig. S7 Consensus around the usability of the FIICCS in football environment

Table S5 Answers on the usability of the FIICCS in football environment

**Domain**	**1**	**2**	**3**	**4**	**5**	**Total**

Contact type - Core set	0	0	0	5	7	12
Physical activity - Core set	0	0	1	5	6	12
Ball situation - Core set	0	0	2	4	6	12
Session detail - Core set	0	0	2	4	6	12
Contact type - Optional set	0	0	1	2	6	9
Physical activity - Optional set	0	0	1	3	6	10
Ball situation - Optional set	0	0	3	2	5	10
Session detail - Optional set	0	0	2	3	5	10
Contextual information - Optional set	0	1	2	5	2	10

1 = Very difficult; 2 = Difficult; 3 = Neutral; 4 = Easy; 5 = Very easy
Total number of panellists included in this round = 12. The difference between the total number of panellists and the total number of answers provided represents the number of answers not given for each question

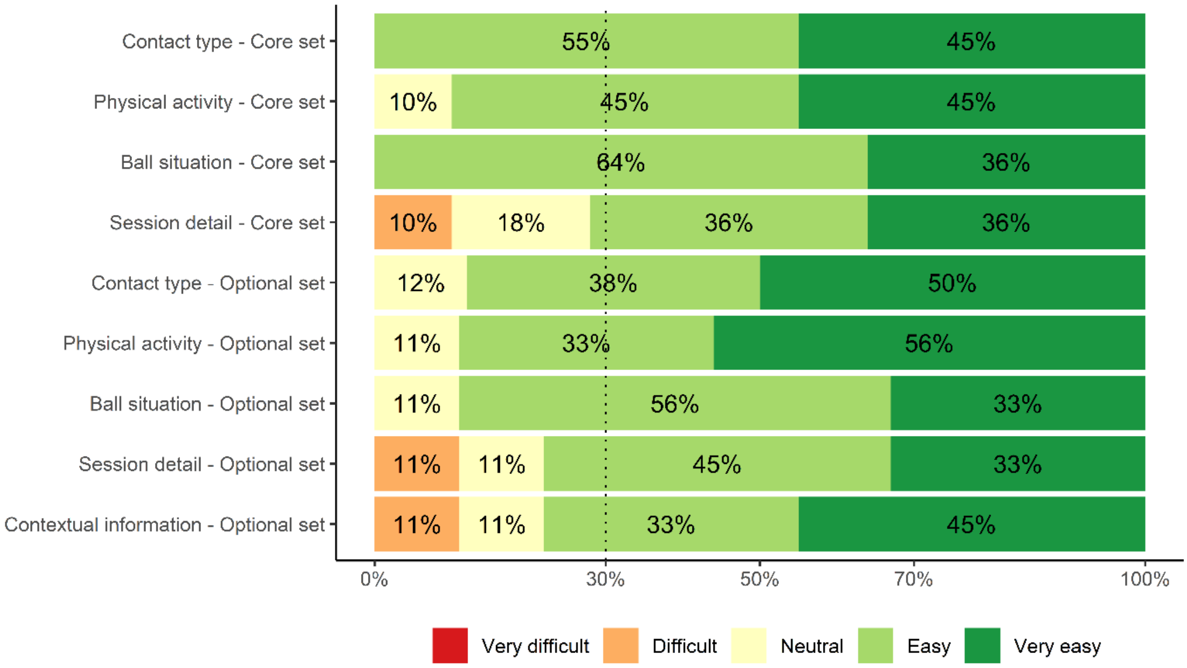

Fig. S8 Consensus around the usability of the FIICCS in research environment

Table S6 Answers on the usability of the FIICCS in research environment

**Domain**	**1**	**2**	**3**	**4**	**5**	**Total**
Contact type - Core set	0	0	0	6	5	11

Physical activity - Core set	0	0	1	5	5	11
Ball situation - Core set	0	0	0	7	4	11
Session detail - Core set	0	1	2	4	4	11
Contact type - Optional set	0	0	1	3	4	8
Physical activity - Optional set	0	0	1	3	5	9
Ball situation - Optional set	0	0	1	5	3	9
Session detail - Optional set	0	1	1	4	3	9
Contextual information - Optional set	0	1	1	3	4	9

1 = Very difficult; 2 = Difficult; 3 = Neutral; 4 = Easy; 5 = Very easy
Total number of panellists included in this round = 12. The difference between the total number of panellists and the total number of answers provided represents the number of answers not given for each question

Table S7 Answers to the pre-meeting survey (phase three)

**Question**	**1**	**2**	**3**	**4**	**5**	**Total**
Reduce the number of questions included in the core set	1	4	3	2	1	11
Make all questions belonging to core set	1	2	2	4	2	11
In question 1 add one more option (reported by medical)	0	2	1	5	3	11
Add a question to ask how many days after the injury the form was filled out	0	2	0	5	4	11
Rephrase “acute” and “overuse” with “sudden onset” and “gradual onset”	0	1	2	5	3	11
In “Contact type” include the option "unclear"	0	1	1	5	4	11
In “Type of contact” include "collision"	0	1	1	6	3	11

In “Tackle direction”, restructure the answers	0	0	3	6	3	11
In “Physical activity – running activity” include “curved run”	0	1	2	7	1	11
Remove controlling phase	0	1	1	7	1	11
Ball situation: add “ball being contested for”	0	1	2	7	1	11
Remove pitch condition	0	3	3	5	0	11
Include pitch condition in core set	0	3	5	3	0	11
Include additional options for referee decision	0	0	3	7	1	11
Remove player attention	0	2	1	5	3	11
Remove where was the player when the injury occurred	1	1	3	5	1	11
Change playing position questions	0	1	6	4	0	11
Include option “Not possible to determine/unclear”	0	1	0	5	5	11
Include a guideline/user manual	0	0	0	7	4	11
Add question for change of direction to indicate the side injured	1	1	3	6	0	11
Running intensity should be discussed during the meeting for alternatives	0	1	1	8	1	11
Ball situation: include a question for player error	1	1	6	2	1	11

Add a question to specify ball impact in kicking - player activity	1	0	5	3	2	11
Include sub-phases for goalkeepers in playing sub-phase	0	1	6	4	0	11
Include “other” option in playing sub-phase	0	0	3	8	0	11
Include own team’s current ranking	1	2	4	3	1	11
Include opponent teams ranking	1	2	4	3	1	11
Include phase of season	1	1	3	2	4	11
Include time of match/training of injury	0	0	1	4	6	11
Specify how many minutes the injured player played	1	0	0	3	7	11

1 = Strongly disagree; 2 = Disagree; 3 = Neither agree nor disagree; 4 = Agree; 5 = Strongly agree
Total number of panellists included in this round = 12. The difference between the total number of panellists and the total number of answers provided represents the number of answers not given for each question

Table S8 Topics discussed during the online meeting (phase four)

**Question N.**	**Question**
1	What is the best length of the core set?
2	After the changes made, what is the clarity of the section ball situation – optional set?
3	How should running intensity be reported?
4	When injuries occur during changes of direction, should we indicate if the injured leg was the one used to change direction?

5	Shall we include a question to indicate whether the injury occurred after a player error?
6	When injuries occur during kicking, shall we specify the type of kick performed (e.g., volley, sidekick)?
7	Shall we remove the question to report pitch condition?
8	Shall we remove the question to report player position on pitch at time of injury?
9	How shall we report playing position of the injured player?
10	Shall we indicate own and opponent teams ranking position?
11	Shall we indicate the phase of season at time of injury?
12	Shall we include further option to report goalkeeper-specific activities?

Table S9 Answers following meeting discussion

**Question**	**1**	**2**	**4**	**5**	**Prefer not to say**	**Total**
Keep the number of questions in the core set as originally proposed			9		1	10
After the changes made, what is the clarity of section "ball situation"?		1	7	1	1	10
Running intensity should be reported as originally proposed		1	6	2	1	10
Include a question to indicate which leg had contact with the ground when the	1		3	5	1	10

injury occurred during a change of direction						
Include a question to indicate whether the injury occurred after a player error	4	4		1	1	10
Add a question to specify the type of kick during which injury occurred		1	5	2	2	10
Include question on "pitch condition" in the classification system		7	2	1		10
Remove question on "player position on pitch at time of injury"	1	1	2	5	1	10
Report only natural position of player and exclude "actual" playing position		2	6		2	10
Include a question for own teams and opponent team ranking position	3	6			1	10
Include a question to indicate the phase of season during which the injury occurred		8	1		1	10
Include question to further specify goalkeeper sub-phases		4	3	1	2	10

1 = Strongly disagree; 2 = Disagree; 4 = Agree; 5 = Strongly agree
Total number of panellists included in this round = 12. The difference between the total number of panellists and the total number of answers provided represents the number of answers not given for each question.

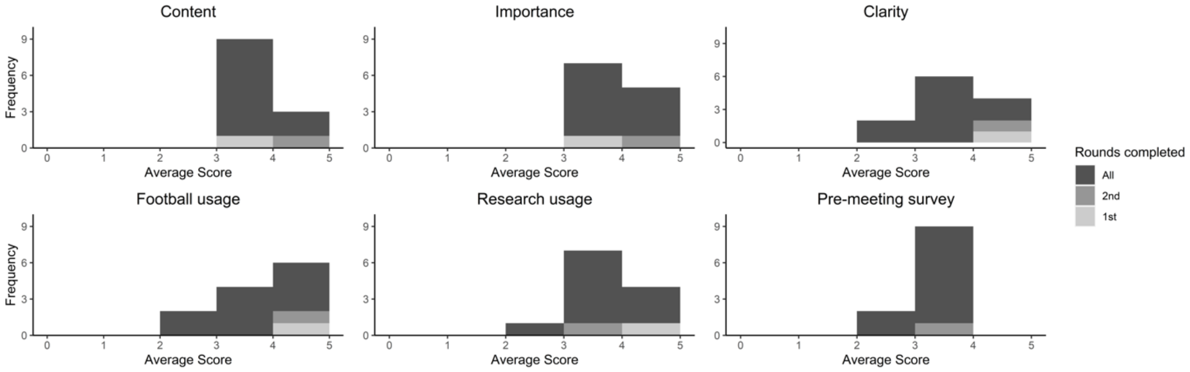

Fig. S9 Average scores in phase two by question category and in phase three. All are panellists who completed the study, second is the panellist who completed phase two and three but did not attend the panel discussion, first is the panellist who completed only phase two
